# Transcriptomic analysis of long noncoding RNAs and mRNAs expression profiles in the spinal cord of bone cancer pain rats

**DOI:** 10.1186/s13041-020-00589-2

**Published:** 2020-03-24

**Authors:** Xinran Hou, Yingqi Weng, Qulian Guo, Zhuofeng Ding, Jian Wang, Jiajia Dai, Anqi Wei, Zongbin Song

**Affiliations:** Department of Anesthesiology, Xiangya Hospital, Central South University, Changsha, Hunan 410008 People’s Republic of China

**Keywords:** Bone cancer pain, Long noncoding RNA, mRNA, High-throughput RNA sequencing

## Abstract

Bone cancer pain (BCP) is one of the most common types of chronic cancer pain and its pathogenesis has not been fully understood. Long non-coding RNAs (lncRNAs) are new promising targets in the field of pain research, however, their involvements in BCP have not been reported. In the present study, we established the BCP model by implantation of Walker 256 carcinoma cells into rats’ tibial medullary cavity and performed transcriptome sequencing of the ipsilateral lumbar spinal cord to explore changes in expression profiles of lncRNA and mRNA. We identified 1220 differently expressed mRNAs (DEmRNAs) (1171 up-regulated and 49 down-regulated) and 323 differently expressed lncRNAs (DElncRNAs) (246 up-regulated and 77 down-regulated) in BCP model, among which 10 DEmRNAs (5 up-regulated and 5 down-regulated) and 10 DElncRNAs (5 up-regulated and 5 down-regulated) were validated the expression by RT-qPCR. Then, we performed Gene Ontology (GO) and Kyoto Encyclopedia of Genes and Genomes (KEGG) analysis on the expression of DEmRNAs and DElncRNAs, showing that they were mainly enriched in inflammatory and immunologic processes/pathways. Finally, we constructed a co-expression network and a ceRNA network of DEmRNAs and DElncRNAs to exhibit a potential regulatory mechanism of DElncRNAs, directly regulating protein coding gene expression *in cis* or *in trans* and indirectly regulating protein coding gene expression by sponging miRNA. In conclusion, our study provided a landscape of dysregulated lncRNA and mRNA in spinal cord of bone cancer pain and detected novel potential targets for treatment in the future.

## Introduction

Cancer pain, caused by primary cancer itself or metastases [1], often deteriorates the quality of life of cancer patients, with a prevalence rate of 50.7% in all cancer stages and 66.4% in advanced stage [2]. Bone cancer pain (BCP), in which the majority is caused by metastatic tumors, is the most common type of cancer pain [1]. Clinically, BCP is one of the most intractable pain, with its complex manifestation such as ongoing pain and breakthrough pain [3]. As many as 70% of breast or prostate cancer patients with skeletal metastases would subsequently develop into BCP [4]. Opioid analgesics were considered as the golden standard in the treatment of cancer-related pain, but nonmedical opioid use and potential social disorder in cancer patients complicate the situation [5], besides the direct adverse reactions of opioids. It is of great clinical demand to clarify the underlying mechanism of BCP, thus determining new potential therapeutic targets.

The development of BCP is accompanied by changes in numerous genes expression in the peripheral and central nervous system, which may account for this dysfunctional nociceptive perception [6–8]. Our previous studies have shown that dysregulated HDACs and its potential downstream targets in the lumbar spinal cord contribute to the development of BCP [9, 10]. In fact, endogenous noncoding RNAs are another key regulators to modify the expression of mRNAs or protein targets, thereby contributing to the development of pathologic pain [11].

Long non-coding RNA (lncRNA) is a class of non-coding RNAs with sequence length greater than 200 nucleotides. LncRNAs are proved to be able to regulate the expression of ion channel related genes [12], purinergic receptor related genes [13], etc. in neuropathic pain. Although the pivotal role of lncRNA in neuropathic pain has attracted extensive attention [14, 15], the involvement of lncRNA in BCP pathogenesis has rarely been explored.

Thus, in the present study, we performed transcriptome sequencing of ipsilateral lumbar spinal cord of BCP rats, induced by Walker 256 carcinoma cells inoculation into tibia cavity, to explore changes in expression profiles of lncRNA and mRNA, and functional analysis of differential genes to provide new insights on the mechanism of BCP.

## Materials and methods

### Experimental animals

Female Wistar rats from the experimental animal center of Central South University (Changsha, Hunan, China), were used in all animal experiments. Rats were group housed in clear plastic cages with sawdust bedding with room temperature at 22 ± 2 °C, light/dark cycle of 12/12 h and freely accessible to food and water. All efforts were made to minimize suffering and to reduce the number of animals used to the minimum required for statistical accuracy. All experimental procedures were approved by the Animal Committee of Xiangya Hospital, Central South University and were performed in accordance with the guidelines of the International Association for the Study of Pain (IASP).

### Modeling of bone cancer pain

The rat model of BCP was established by implantation of Walker 256 carcinoma cells into the medullary cavity of the tibia as we previously described [9]. Briefly, Walker 256 breast cancer cell suspension (Dingguo Changsheng, China), syngeneic to Wistar rats, was thawed, rinsed, and then centrifuged at 1000 rpm for 5 min, followed by intraperitoneal inoculation in Wistar rats (weighed 80–100 g). 7–10 days later, malignant ascites was extracted, rinsed, centrifuged and diluted into 1 × 10^7^ cells/mL for inoculation.

Adult Wistar rats (weighed 220–250 g) were anesthetized with intraperitoneal injection of 50 mg/kg phenobarbital sodium. After shaved and disinfected, a 1 cm transverse superficial incision was made in the skin overlying ligamentum patellae, then a 22-gauge needle was used to perforate the bone cortex with entry point at intercondylar eminence. After removal of puncture needle, a 50-μL microsyringe was inserted into the needle hole and 10 μL of Walker 256 cell suspension was injected into the medullary cavity of tibia slowly, while sham-operated rats were injected with heat-killed cancer cells instead. The puncture point was sealed with bone wax and the incision was irrigated with normal saline and interruptedly sutured.

### Radiological and pathological examination of the tibia

To verify the bone destruction caused by tumor inoculation, X-ray radiography image of the left hind limb was obtained at 14 days after BCP modeling. In addition, the left tibia bone tissue was harvested, preserved in 4% paraformaldehyde and decalcified in 10% EDTA for another 21 days. Finally, the tibia was embedded in paraffin, sectioned into 5-μm-thick slice and stained with hematoxylin and eosin (H&E).

### Behavioral test

Mechanical hyperalgesia was measured by von Frey filaments as previously described [9]. In brief, each rat was allowed to acclimate to a Plexiglas box (22 × 12 × 12 cm^3^) with a wire mesh floor for 30 min before test; then, a series of calibrated blunt-pointed von Frey filaments with logarithmically incremental stiffness (ranging from 0.6 g to 26 g) were stabbed perpendicularly against the plantar surface of hind paw with even force. A positive response was defined as rapid paw withdrawal and/or paw flinching accompanied by head turning, biting, or licking upon application of von Frey filament. The incremental von Frey filament would be applied until a positive response aroused. The paw withdrawal mechanical threshold (PWMT) was recorded as the lowest force evoking three positive responses out of five applications. Each rat was tested three times and the data were averaged with the experimenter blind to animal grouping.

### Tissue collection and RNA extraction

On the 14th day posterior to BCP modeling, all rats were deeply anesthetized, followed by decapitation and exsanguination. The lumbar enlargement spinal cord was quickly isolated out, removed the meninges, and dissected longitudinally on ice; then the ipsilateral spinal cord was snap-frozen and preserved in liquid nitrogen. Total RNA was isolated using RNeasy mini kit (Qiagen, Germany) and checked for a RIN (RNA Integrity Number) to inspect RNA integrity by an Agilent Bioanalyzer 2100 (Agilent technologies, USA), followed by purified by RNAClean XP Kit (Beckman Coulter, USA) and RNase-Free DNase Set (QIAGEN, Germany) according to manufacturers’ instructions, respectively.

### Construction of cDNA libraries and high-throughput sequencing

Strand specific libraries were prepared using the TruSeq® Stranded Total RNA Sample Preparation kit (Illumina, USA). Briefly, ribosomal RNA was removed from total RNA by Ribo-Zero rRNA removal beads. Then fragmented RNA was copied into first strand cDNA using reverse transcriptase, followed by second strand cDNA synthesis using DNA Polymerase I and RNase H. After undergoing an end repair process, the addition of a single ‘A’ base, and then ligation of the adapters, cDNA fragments were purified and enriched with PCR to create the final cDNA library followed by quantified with Qubit® 2.0 Fluorometer (Life Technologies, USA) and validated by Agilent 2100 bioanalyzer (Agilent Technologies, USA) to confirm the insert size and calculate the concentration. Cluster was generated by cBot with the library diluted into 10 pM and sequenced on the Illumina HiSeq X-ten (Illumina, USA). The library construction and sequencing were performed at Shanghai Biotechnology Corporation.

### Quantification of gene expression and differential expression analysis

Sequencing raw reads were filtered by Seqtk followed by mapping to the rat Rnor_6.0 reference genome using Hisat2 (version:2.0.4) [16]. Stringtie (version:1.3.0) [17, 18] was used to calculate Fragments Per Kilobase of exon model per Million mapped reads (FPKM) with a reference annotation, then edgeR was used to identify differentially expressed mRNAs (DEmRNAs) and differentially expressed lncRNAs (DElncRNAs) [19], with filter criteria set as *P* < 0.05, false discovery rate (FDR) < 0.05 [20] and fold change (FC) > 2. Gffcompare (version: 0.9.8) was used to predict novel lncRNA not included in NONCODE v5 [21] and Ensembl database. The location of total identified mRNAs and lncRNAs were schematized on rat chromosome karyotype, among which DEmRNAs and DEmRNAs were indicated, with the use of Idiographica [22].

### Real-time quantitative PCR (RT-qPCR)

Total RNA was extracted from the spinal cord sample, purified and quality inspected ibidem. Then, cDNA was synthesized with ReverTra Ace qPCR Kit (TOYOBO, FSQ-101, Japan) according to the manufacturer’s instructions. Real-time fluorescent quantitative PCR was performed on QuantStudio 5 Real-Time PCR System (Applied Biosystems, USA) with total volume of 20 μl containing 10 μl 2X SYBR Green PCR buffer (ABI, 4368708, USA), 1 μl PCR forward primer (10 μM), 1 μl PCR reverse primer (10 μM), 2 μl template and 6 μl nuclease-free water in each reaction. Three-step program for PCR reaction by QuantStudio™ Design & Analysis Software was as follows: 1 cycle for initial denaturation at 50 °C for 2 min and 95 °C for 10 min, then 40 cycles for PCR amplification with denaturation at 95 °C for 15 s, annealing and extension at 60 °C for 1 min, followed by a melting curve analysis. All experiments included no-template controls and all samples were analyzed independently in triplicate. Expression data were normalized to the expression of Gapdh with 2^-ΔΔCt^ method. The sequences of the used primers were listed in Table 1.

**Table 1:** Sequences of primers in RT-qPCR

Gene name	Primer sequences (5′-3′)
Cybb	Forward: ACCCAGATGCAAGAAAGAAACAA
	Reverse: GCAAAGTGATTGGCCTGAGATT
CD74	Forward: CATCTAAGGGACCCCCATTTC
	Reverse: CCTAGATCTCAGAGCCCCACAT
Trpm2	Forward: GAACCAGGGTGGAGGCATCT
	Reverse: TGACCAGCACCTCCAACATC
Ciita	Forward: TGAGCACCAGACAGTGGAGTGT
	Reverse: GAGATGCTCCCAGGATGCA
Anxa3	Forward: CCATGGGAGGGAGAGACAGA
	Reverse: CTGAAGTATCCTCTCCTCCTTTGTG
NONRATT003582.2	Forward: AAATTCTTTAGCTCAGCCCTGCTA
	Reverse: AATGCACGGATCCCATCTTAA
NONRATT004661.2	Forward: GTATTGCGAACAAAGTCCATCCT
	Reverse: GCATCCGCGAGCATGTG
NONRATT007487.2	Forward: AGCCAGTGGGTCACTTGTGTT
	Reverse: TTCTGCTAAAGTCCGGCTGAA
NONRATT026544.2	Forward: CCCTCGTCCCTCTGGTCTATG
	Reverse: GGCTTGCTCAGGGATGCA
NONRATT008764.2	Forward: GAAGAGTGGGCCGAGAACAA
	Reverse: AGGCAGACATCAGACACTGC
Crlf1	Forward: TGCCGGCTAAACTCTGAGGAT
	Reverse: GTGAGCCTCCAGGTCTGCAT
Cdc42ep2	Forward: CCCGCCTCCTCTCCAACTAC
	Reverse: GACCGGTGGGAAGCATACC
Hspa1b	Forward: TGCATGTTCTTTGCGTTTAATCTAA
	Reverse: AGGTGTTCGCAGGAAGGAAA
AABR07031184.1	Forward: TGTCCTTTCACGTGCTTGCT
	Reverse: AGGTGAAAGGCTAAGGCAGACTT
Tnnc2	Forward: CGCATTGACTTCGACGAGTTT
	Reverse: GAACACGAAGACCAGCTCCTTAC
MSTRG.12616.2	Forward: TTCCCAGGCAGCTTCAGACT
	Reverse: AGCGGGTTGGGTCATCAG
MSTRG.13351.2	Forward: CACATTGTATATCTGCGCCACAT
	Reverse: TCAGCCTAGATGCCCAAGAAC
MSTRG.16194.3	Forward: CCAGTGTCAAATGCATGCTCAT
	Reverse: CCTGGTCTCTCCCGCTAGAA
MSTRG.16806.2	Forward: GAAGCGCTTAAGCAAAGTCTCTTC
	Reverse: CGATACTTTCACCAAACTCTTCCTT
MSTRG.29385.15	Forward: TCCACTGGCTCAATCATGTGA
	Reverse: TCCCCAGAAACAGGGATCAA
Gapdh	Forward: TGGCCTCCAAGGAGTAAGAAAC
	Reverse: GGCCTCTCTCTTGCTCTCAGTATC

### Target prediction of DElncRNAs

LncRNA could regulate gene expression *in cis* and *in trans* manner. The potential target coding genes *in trans* were predicted by the sequence similarity of lncRNA and mRNA with BLAST and their complementary energy, which is above the threshold, computed by RNAplex [23]; while the coding genes transcribed within 10 kb upstream or downstream of lncRNA in genomic localization were considered as potential target genes *in cis*.

### Gene ontology (GO) and Kyoto encyclopedia of genes and genomes (KEGG) enrichment analysis

GO analysis for DEmRNAs and targets of DElncRNAs was performed to construct gene annotations from the perspective of biological process, cellular component and molecular function. GO analysis was accomplished by DAVID Bioinformatics Resources 6.8 with *P* values and FDR used to test the reliability of the analysis [24] and the top 10 terms (according to P value) in each class were presented in bar graph. KEGG pathway analysis was performed to understand the function and interactions among differentially expressed genes and the top 30 pathways were presented in bubble diagram. The enrichment factor was the value ratio between the sequenced gene and all annotated genes enriched in the pathway.

### Cellular expression analysis of DEmRNA and comparative analysis, protein-protein interaction (PPI) network construction and functional enrichment of top 200 DEmRNAs

Based on the expression data reported by Ye Zhang et al. [25], we constructed expression heatmap of DEmRNA among 7 types of cells (i.e., neuron, microglia, astrocytes, oligodendrocyte precursor cell, newly formed oligodendrocyte, myelinating oligodendrocytes, and endothelial cells). The top 200 DEmRNAs (according to *P* value) were selected to compare with the results of another two researches focused on gene expression variation in the spinal cord in rats chronic pain model, with one by microarray assay [26] and the other by RNA-seq assay [27]. The comparative analyses of three datasets were performed by a web-based portal, Metascape [28]. The PPI network between the top 200 DEmRNA-encoded protein was constructed by the online database STRING (v11.0, http://string-db.org/) and visualized by Cytoscape 3.7.1 [29] and was further analyzed by Molecular Complex Detection (MCODE) [30] clustering algorithm to construct subnetwork. The GO biological process and KEGG pathway enrichment of the top 200 DEmRNA were performed and visualized by Metascape [28].

### Co-expression analysis of DElncRNAs/mRNAs

The Pearson correlation coefficient (PCC) of DElncRNAs and DEmRNAs expression value was calculated, and |PCC| > 0.99 and *P* < 0.001 was set as filter to construct co-expression network of DElncRNAs and DEmRNAs, however, there were too many nodes to exhibit. So, we took the intersection of DEmRNAs and target mRNA of DElncRNAs, and constructed a co-expression network of DElncRNAs and the intersectional DEmRNAs and Cytoscape 3.7.1 was used to exhibit the network [29].

### Competing endogenous RNA (ceRNA) analysis of DElncRNAs and DEmRNAs

The possible target binding of lncRNA/mRNA and miRNA was predicted by miRanda, with a maximum binding free energy less than − 20 [31]. In consideration of the scale of the figure, we set the threshold of |PCC| of relative expression value of DElncRNAs and DEmRNAs more than 0.98 to exhibit the network and Cytoscape 3.7.1 was used to exhibit the network [29].

### Statistical analysis

The behavioral test and RT-qPCR data were presented as the mean ± standard deviation (SD). For behavioral test data, repeated measures ANOVA was performed to detect overall differences followed by Bonferroni post hoc analysis for multiple comparisons. For RT-qPCR data, student’s t-test was used to determine the differences between groups. *P* < 0.05 was regarded as statistically significant. GraphPad Prism 8 Software (Graph-Pad, USA) was used for all statistical analyses and statistical graph drawing.

## Results

### Rats developed mechanical hypersensitivity and osteolytic lesions after tumor cells inoculation

**Fig. 1:**
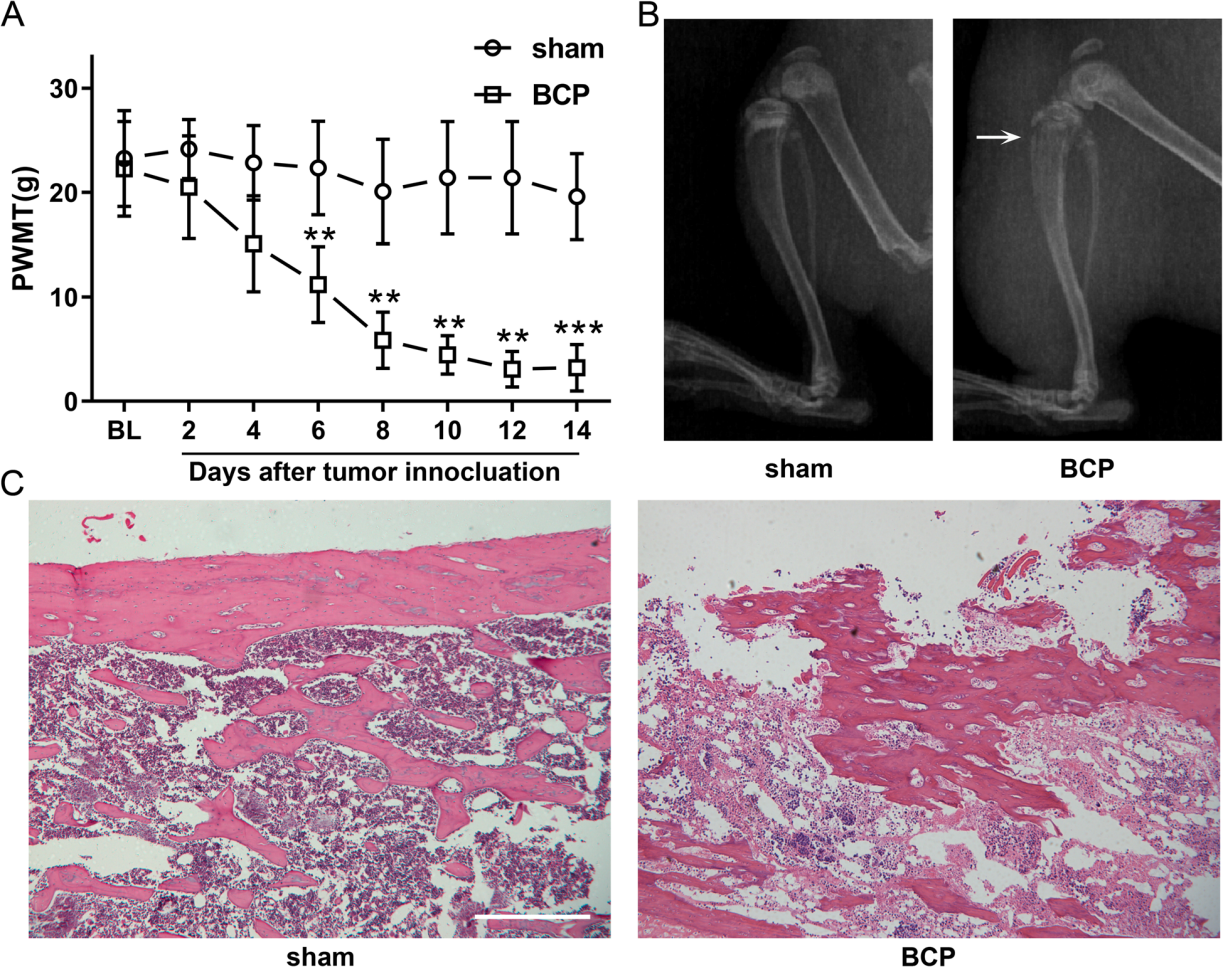
Intratibia inoculation of Walker 256 carcinoma cells caused progressive hyperalgesia and bone destruction. **a**. Mechanical hypersensitivity arose from 6 days after modeling and lasted to the end of behavioral test, while the mechanical threshold of sham-operated rats kept stable. The data are presented as the mean ± SD, **P < 0.01, ***P < 0.001 compared with sham-operated rats at each timepoint; *n* = 6, per group. PWMT, paw withdrawal mechanical threshold; BL, baseline; sham, sham-operated group; BCP, bone cancer pain group. **b**. X-ray examination of left hind paw showed discontinuity and destruction of cortical bone (marked by the arrow) and swelling of soft tissue in proximal tibia of bone cancer pain rats. **c**. Microscopic examination of tibia slices, by HE staining, showed severe destruction and disarrangement of bone trabecula in bone cancer pain rats, scale bar = 500 μm

There was no significant difference in the PWMT baseline between sham-operated rats and BCP rats; the PWMT of BCP rats declined consistently until 10 days after tumor inoculation and maintained at relatively low level, while PWMT of sham rats remained unchanged. The differences in PWMT between the two groups were significant on day 6, 8, 10, 12 and 14 after modeling (*P* < 0.01 and *P* < 0.001 respectively), indicating the development of mechanical hypersensitivity in BCP rats (Fig. 1a, Additional file 1 Table S1). Radiological and pathological examination of tibia bone demonstrated consistently severe cortical bone destruction and disarrangement of bone trabecula in BCP rats compared with sham-operated rats indicating osteolytic lesions after modeling (Fig. 1b, c).

### Transcriptomic high-throughput sequencing revealed changes in gene expression profiles

A total of 506,546,810 raw reads were obtained using the Illumina HiSeq X-ten platform and 485,363,772 clean reads were generated after removing adaptor, short fragment reads and other low-quality sequences with clean ratio more than 95% in all samples. After ribosome RNA reads trimmed, 440,181,683 reads were mapped to the rat Rnor_6.0 reference genome among 479,470,030 reads in all with an average mapping rate of 91.8% (Additional file 2 Table S2). The raw data and processed data have been deposited in the National Center for Biotechnology Information’s Gene Expression Omnibus (GEO), with the accession number GSE137326.

**Fig. 2:**
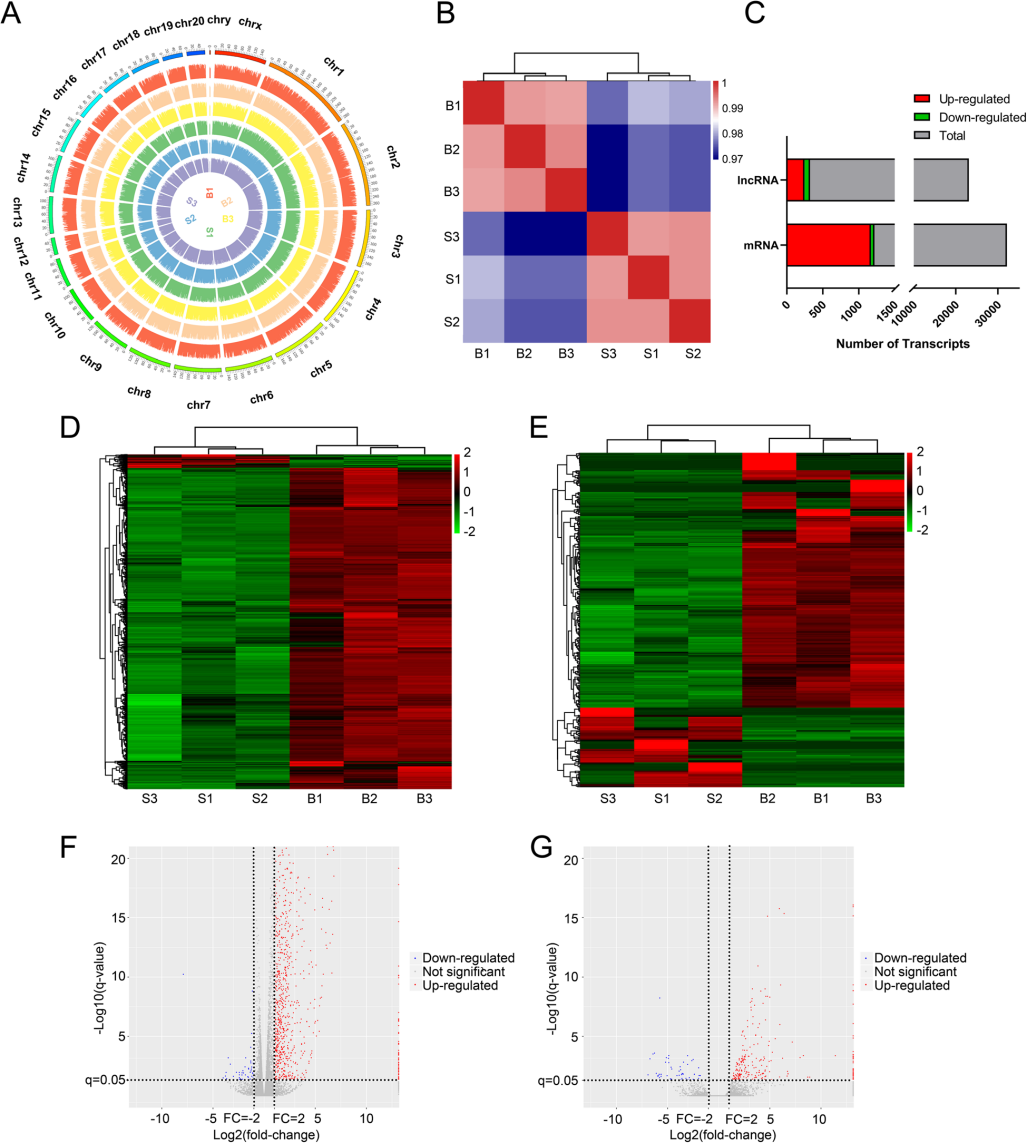
Tumor inoculation caused significant changes in mRNA and lncRNA profile detected by transcriptomic high-throughput sequencing. **a**. Circos plot of genome coverage showed the detected reads were distributed in all chromosomes, similarly in each sample. S1, S2, S3, triplicate sample of the sham-operated group; B1, B2, B3, triplicate sample of bone cancer pain group. **b**. Heatmap of gene expression correlation between each sample. Pearson’s correlation coefficient within the same biological group was high, indicating intra-group consistency, and the coefficient in different groups was low, indicating inter-group variance. **c**. Statistic description of detected transcripts overall and differently expressed, respectively. **d**. Hierarchical cluster analysis of differently expressed mRNA (DEmRNA). The color scale indicates log_10_FPKM and intensity increases from green to red, which indicates down- and up-regulation, respectively. **e**. Hierarchical cluster analysis of differently expressed lncRNA (DElncRNA). ***f****. volcano* plot of DEmRNA, in which vertical lines correspond to 2-fold changes in upregulation or downregulation; horizontal line represents q = 0.05 (*p* value adjusted by FDR); red points refer to up-regulated mRNAs and blue points refer to down-regulated mRNAs. **g**. Volcano plot of DElncRNAs

In each sample, the gross transcripts were found to be almost equally distributed on all chromosomes (Fig. 2a). Pearson’s correlation coefficient of whole transcripts expression levels among all samples were calculated and visualized by heatmap of inter-sample correlation, demonstrating the obvious difference between BCP and sham-operated rats, but slight difference within each group (Fig. 2b). In total, 30,957 mRNAs and 22,783 lncRNAs were identified. With the filtering criteria of *P* < 0.05, FDR < 0.05 and fold change > 2, 1220 differently expressed mRNAs (DEmRNAs) (1171 up-regulated and 49 down-regulated) and 323 differently expressed lncRNAs (DElncRNAs) (246 up-regulated and 77 down-regulated) were detected after quantification of gene expression and comparison between the two groups (Fig. 2c). Hierarchical clustering of the expression of mRNA and lncRNA showed obvious discrimination between BCP and sham-operated rats (Fig. 2d, e). The count and fold change of DEmRNAs and DElncRNAs were visualized by volcano plot (Fig. 2f, g) and the detailed information of top 20 up-regulated and top 20 down-regulated mRNAs and lncRNAs were listed in Table 2 and Table 3. The total identified mRNAs/DEmRNA and lncRNAs/DElncRNA covered location of all chromosomes (Additional file 3 Figure S1 and Additional file 4 Figure S2). These results indicated that tumor inoculation in tibia shifted expression profiles of lncRNA and mRNA in the ipsilateral lumbar spinal cord, which may account for the development of hyperalgesia of BCP rats.

**Table 2:** The detailed information of top 20 up-regulated and top 20 down-regulated mRNAs

gene name	description	log_2_FC	*P* value
Up-regulated			
RT1-Ba	RT1 class II, locus Ba	6.81	0
Cd74	Cd74 molecule, major histocompatibility complex, class II invariant chain	6.23	0
RT1-Bb	RT1 class II, locus Bb	6.82	0
RT1-Da	RT1 class II, locus Da	6.79	0
Cxcl13	chemokine (C-X-C motif) ligand 13	8.98	3.55E-254
RT1-Db1	RT1 class II, locus Db1	4.74	1.35E-232
Itgax	integrin, alpha X	6.15	3.62E-200
C3	complement component 3	2.98	2.94E-196
Ciita	class II, major histocompatibility complex, transactivator	8.39	2.44E-191
Ncf1	neutrophil cytosolic factor 1	3.65	8.10E-173
Lyz2	lysozyme 2	3.92	1.48E-161
Cxcl16	chemokine (C-X-C motif) ligand 16	3.88	1.53E-160
Clec7a	C-type lectin domain family 7 member A transcript variant 1; Dectin-1; Protein Clec7a	6.85	4.13E-156
Irf8	interferon regulatory factor 8	3.01	7.18E-139
Scin	scinderin	> 10	7.22E-137
Cxcl9	chemokine (C-X-C motif) ligand 9	5.75	5.15E-135
Plek	pleckstrin	3.04	5.72E-132
Ubd	ubiquitin D	6.71	3.27E-131
Ptprc	protein tyrosine phosphatase, receptor type, C	3.46	4.64E-123
Gpnmb	glycoprotein (transmembrane) nmb	2.88	5.98E-123
Down-regulated			
AABR07031184.1	–	−7.84	2.04E-12
Crlf1	cytokine receptor-like factor 1	−1.10	6.20E-11
Hspa1b	heat shock protein 1B	−1.17	3.59E-07
Tnnc2	troponin C type 2 (fast)	−1.26	6.34E-06
F12	coagulation factor XII (Hageman factor)	−3.45	6.14E-05
Mesp2	mesoderm posterior basic helix-loop-helix transcription factor 2	−1.74	6.18E-05
Hif3a	hypoxia inducible factor 3, alpha subunit	−1.25	0.000125375
Hpn	hepsin	−1.25	0.000186518
Agtr1a	angiotensin II receptor, type 1a	−1.01	0.000198987
Slc7a15	solute carrier family 7 (cationic amino acid transporter, y + system), member 15	−3.25	0.000249069
Ascl2	achaete-scute family bHLH transcription factor 2	−1.81	0.00045278
Cdc42ep2	CDC42 effector protein (Rho GTPase binding) 2	−1.15	0.000468742
Alb	albumin	−1.47	0.000614844
Cfap46	cilia and flagella associated protein 46	−1.46	0.000737338
AC096430.2	–	−1.19	0.000745128
AABR07069524.1	–	−2.02	0.00105327
Ripply2	ripply transcriptional repressor 2	−1.22	0.001084151
Ucn	urocortin	<−10	0.001361414
Eln	elastin	−1.31	0.00150543
7SK	7SK RNA	−2.66	0.001521089

**Table 3:** The detailed information of top 20 up-regulated and top 20 down-regulated lncRNAs

LncRNA id	Chromosomal locus	Log_2_FC	*P* value
Up-regulated			
NONRATT029597.2	9:92704937–92718218	> 10	1.13E-20
NONRATT025792.2	7:54213332–54236613	> 10	2.12E-20
NONRATT013483.2	18:56078109–56079084	5.98	3.89E-20
NONRATT013481.2	18:56076008–56076835	6.45	1.40E-19
NONRATT025583.2	7:12490976–12493364	> 10	2.51E-19
NONRATT020861.2	4:152968621–152969931	4.83	3.01E-19
NONRATT008764.2	13:55114409–55116581	3.89	5.05E-15
NONRATT009773.2	14:15253248–15258192	> 10	2.17E-13
NONRATT023930.2	5:172329170–172330108	6.11	2.45E-13
MSTRG.29606.1	8:58637292–58641625	3.35	5.65E-13
NONRATT008238.2	13:51970515–51971438	4.59	6.14E-13
NONRATT013482.2	18:56077434–56080851	> 10	9.18E-13
NONRATT019710.2	3:124458778–124460981	> 10	1.00E-12
NONRATT001368.2	1:215660363–215662507	4.17	1.14E-12
NONRATT022734.2	5:153532201–153533094	4.82	3.52E-12
NONRATT026079.2	7:118911458–118913673	4.66	3.52E-12
NONRATT021744.2	4:163393219–163401090	5.05	4.37E-12
NONRATT007487.2	12:38161648–38162391	3.32	6.42E-12
NONRATT004661.2	10:70310274–70312073	3.29	1.11E-11
NONRATT015068.2	2:55981585–55983021	3.14	1.43E-11
Down-regulated			
MSTRG.29385.15	8:45815956–45825522	<−10	2.59E-24
MSTRG.16806.2	2:156857842–156861509	<−10	1.13E-20
MSTRG.19227.2	3:27166506–27169797	<− 10	5.40E-20
MSTRG.16194.3	2:78174449–78184975	<−10	5.40E-12
MSTRG.13351.2	17:58405803–58485207	−5.70	5.53E-10
NONRATT002487.2	1:87959597–87970073	<−10	6.14E-10
NONRATT020414.2	4:71741571–71746773	<−10	5.21E-09
MSTRG.12616.2	16:85722254–85740881	<−10	2.57E-08
MSTRG.26149.3	6:97231481–97233020	<−10	3.66E-08
MSTRG.9921.3	14:66037977–66050713	<−10	3.84E-08
MSTRG.32467.2	X:13336182–13347008	<−10	6.21E-08
MSTRG.11320.2	15:77416838–77420559	<−10	1.37E-07
NONRATT016515.2	2:183503246–183519420	<−10	8.07E-07
NONRATT016788.2	2:211344017–211344442	−6.21	9.85E-07
NONRATT005011.2	10:97680659–97683273	−6.37	1.41E-06
NONRATT005584.2	10:56339278–56341596	−4.82	1.52E-06
NONRATT005012.2	10:97683239–97684709	−2.98	2.52E-06
MSTRG.4193.2	10:9842392–9844018	−5.06	2.83E-06
NONRATT012007.2	16:54083152–54087424	<−10	3.09E-06
MSTRG.4440.2	10:24800650–24814515	−4.85	3.59E-06

### Validation of expression changes of DElncRNAs and DEmRNAs by RT-qPCR assay

**Fig. 3:**
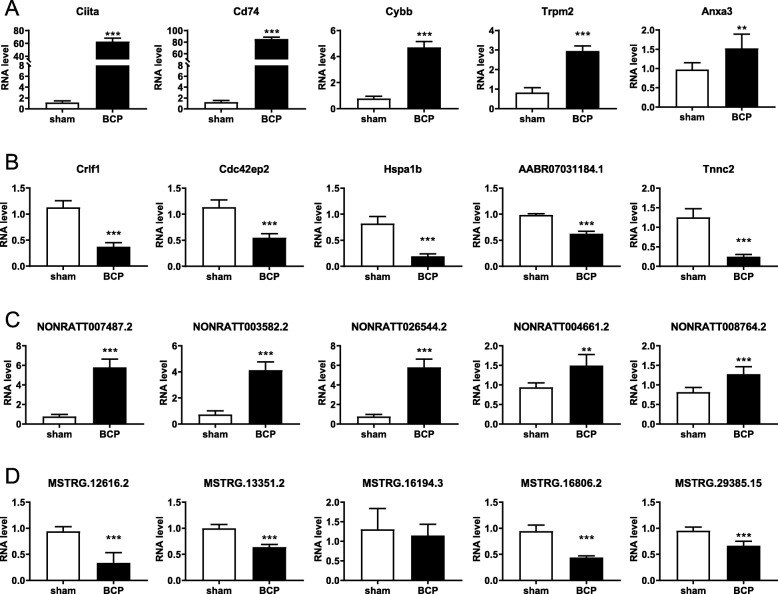
RT-qPCR validation of partial DEmRNAs and DElncRNAs on another batch of samples independent of the ones used for RNA-seq (*n* = 6 per group). **a**. The expression level of up-regulated mRNAs, with gene name on each bar graph. **b**. The expression level of down-regulated mRNAs. **c**. The expression level of up-regulated lncRNAs. **d**. The expression level of down-regulated lncRNAs. **P < 0.01, ***P < 0.001, compared with sham-operated rats

To validate the reliability of sequencing quantification, 10 DEmRNAs (5 up-regulated and 5 down-regulated) and 10 DElncRNAs (5 up-regulated and 5 down-regulated) were selected to undergo RT-qPCR on another batch of samples independent of the ones used for RNA-seq (*n* = 6 per group). As shown in Fig. 3 and Additional file 5 Table S3, quantification by RT-qPCR of all the ten DEmRNAs were consistent with those of RNA sequencing; Class II, major histocompatibility complex, transactivator (Ciita), Cd74, Cytochrome b-245 beta chain (Cybb), Transient receptor potential cation channel, subfamily M, member 2 (Trpm2), annexin A3 (Anxa3) were all significantly up-regulated and AABR07031184.1, cytokine receptor-like factor 1 (Crlf1), heat shock protein 1B (Hspa1b), troponin C type 2 (fast) (Tnnc2), CDC42 effector protein (Rho GTPase binding) 2 (Cdc42ep2) significantly down-regulated. And all the five DElncRNAs, namely NONRATT007487.2, NONRATT003582.2, NONRATT026544.2, NONRATT004661.2, NONRATT008764.2 were up-regulated significantly and four out five DElncRNAs, namely MSTRG.12616.2, MSTRG.13351.2, MSTRG.16806.2, MSTRG.29385.15 were down-regulated, consistent with that of RNA sequencing.

### GO and KEGG analysis of DEmRNAs and DElncRNAs

**Fig. 4:**
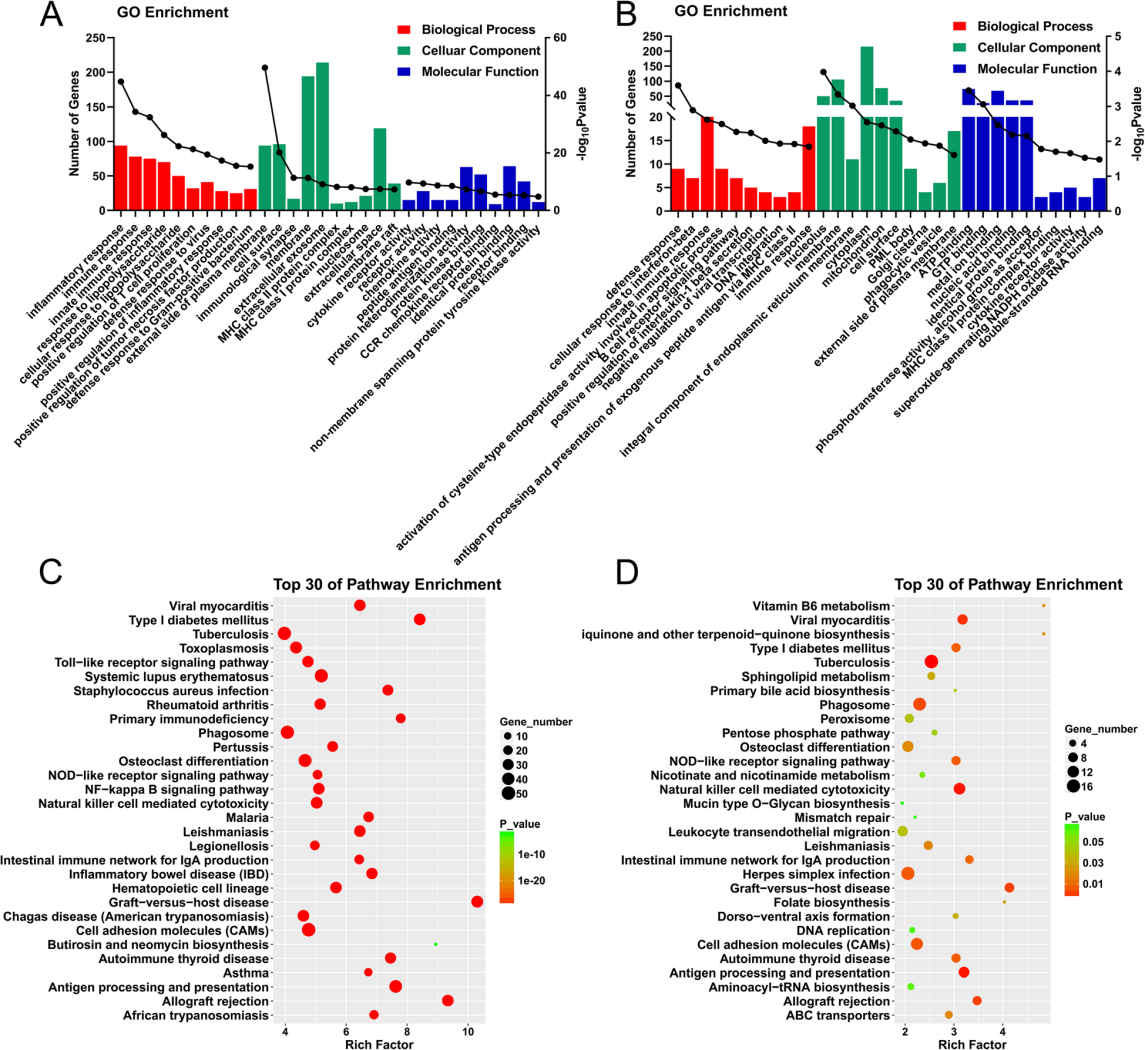
GO enrichment and KEGG enrichment analysis for DEmRNAs and DElncRNAs. **a**. GO enrichment of DEmRNAs showing the top 10 terms (according to *P* value) in three types of GO terms, biological process (shown in red), cellular component (shown in green) and molecular function (shown in blue). **b**. GO enrichment of potential target of DElncRNA. **c**. KEGG enrichment analysis for DEmRNAs showing the top 30 pathways, with the size of bubble indicating the number of enriched mRNAs, the color indicating *p* value and position indicating rich factor. **d**. KEGG enrichment of potential target of DElncRNA

GO enrichment analysis of the DEmRNAs showed that the DEmRNAs were mainly enriched in the following biological process (BP) terms: inflammatory response, immune response, response to lipopolysaccharide, and in the following cellular component (CC) terms: external side of plasma membrane, cell surface, immunological synapse, and in the following molecular function (MF) terms: cytokine receptor activity, receptor activity, chemokine activity, peptide antigen binding (Fig. 4a).

GO analysis of potential target genes of DElncRNAs showed that they were enriched in the following BP terms: defense response, cellular response to interferon-beta, innate immune response, and in the following CC terms: nucleolus, membrane, integral component of endoplasmic reticulum membrane, and in the following MF terms: ATP binding, metal ion binding, nucleic acid binding (Fig. 4b).

Similarly, KEGG analysis of these dysregulated genes showed that neglecting apparently unrelated pathways, DEmRNAs were primarily enriched in allograft rejection, antigen processing and presentation, primary immunodeficiency, etc. (Fig. 4c); and potential target genes of DElncRNAs were primarily enriched in allograft rejection, antigen processing and presentation, natural killer cell mediated cytotoxicity, etc. (Fig. 4d). These results also indicated the involvement of extra−/intra-cellular pathways in inflammatory and immunologic process. The overlap between the GO enrichment and KEGG analysis of DEmRNAs and DElncRNAs suggested that there might be many interactions between DEmRNAs and DElncRNAs in the development of BCP.

### The top 200 DEmRNAs were mainly expressed in microglia and interacted extensively

**Fig. 5:**
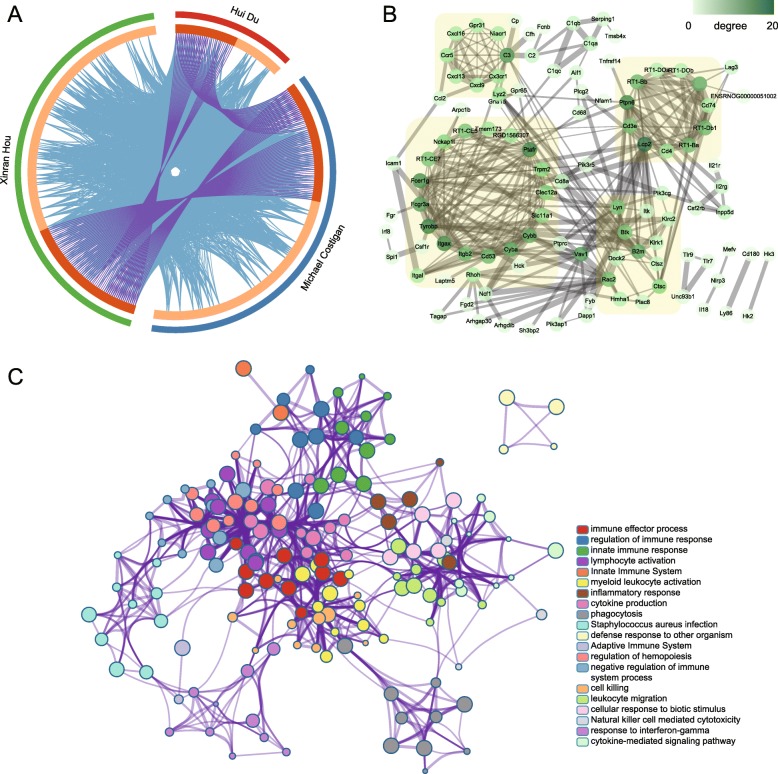
Comparative analysis, Protein-protein interaction (PPI) network and functional enrichment network construction of top 200 DEmRNAs. **a**. The Circos plot showing how genes from the input gene lists overlap, in which on the outside the red arch represents the 58 unique differential genes reported by Hui Du et al., the blue arch representing 173 unique differential genes reported by Michael Costigan et al., and the green arch representing our top 200 unique differential genes; on the inside dark orange color represents the genes that appear in multiple lists and light orange color represents genes that are unique to that gene list; and purple lines link the same genes that are shared by multiple gene lists, blue lines linking the different genes where they fall into the same ontology term (the terms statistically significantly enriched and with size no larger than 100). **b**. PPI network constructed by the top 200 DEmRNA-coding protein. The color scale of the node indicates the number of connectivity with other nodes and the thickness of the edge indicates the combined score of the interaction. The clusters in light yellow frame were the top four subnetworks calculated by MCODE. **c**. Functional enrichment network constructed by nodes representing terms with the best *p* values from each of the 20 clusters, with the constraint that there are no more than 15 terms per cluster and no more than 250 terms in total and edges connecting terms with a similarity > 0.3

As shown in the expression heatmap of DEmRNA among 7 main types of the cells in central nervous system (Additional file 6 Figure S3), the majority of DEmRNAs, especially the top 200, were highly expressed in microglia, in spite of a few exceptions, indicating the critical role of microglial dysfunction in BCP development. By comparison with another two similar researches, 64 DEmRNAs identically overlapped with peer results and much more genes were functionally related (Fig. 5a). PPI network constructed by the top 200 DEmRNA-coding protein showed extensive interaction among them and four subnetworks were clustered, which may represent more compact functional blocks (Fig. 5b). Functional analysis of these genes showed that they were mainly concentrated in immunological processes/pathways, indicating they were highly involved in the pathogenesis of bone cancer (Fig. 5c, Additional file 7 Table S4).

### Co-expression analysis of DElncRNAs/mRNAs

**Fig. 6:**
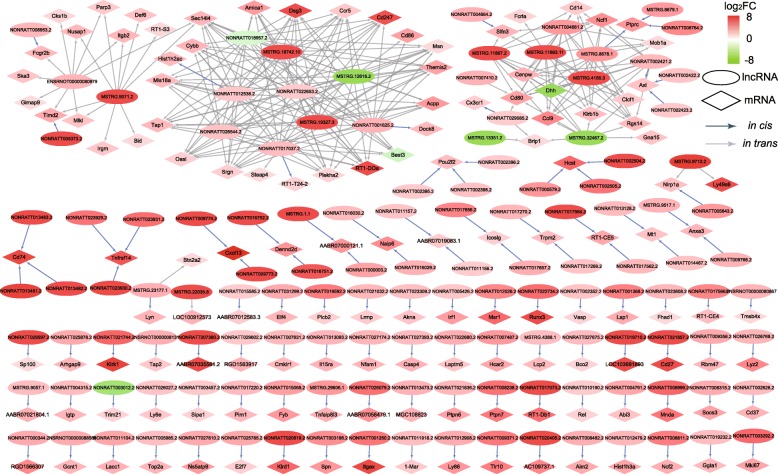
Co-expression network of DElncRNA and their potential target mRNA with 131 lncRNA and 137 mRNA nodes and 350 edges. The color scale indicates log_2_FC, and intensity increases from green to red, which indicates down- and up-regulation, respectively. The ellipse node indicates lncRNA and the rhombus node indicates mRNA. The blue arrow indicates lncRNA regulating mRNA *in cis*, while the gray arrow indicating *in trans*

A delicate co-expression network was constructed based on DElncRNAs and their potential target DEmRNAs, with 131 lncRNAs, 137 mRNAs and 350 edges (Fig. 6). Most of DElncRNAs and their trans-regulated DEmRNAs were interweaved with the hub lncRNAs, such as ENSRNOT00000080979, NONRATT012538.2, MSTRG.12616.2, NONRATT026544.2, NONRATT022653.2, NONRATT001625.2, NONRATT017037.2, and the hub mRNAs, such as Cybb, Tap1, Srgn, Ccr5, RT1-DOa, Msn, Dsg3, CD80, CD247, indicating the involvement of inflammatory and immunologic process, oxidative stress and intercellular communication in BCP. Meanwhile, major DElncRNAs and their *cis*-regulated DEmRNAs were separated pairs, except CD74, Pou2f2, Cxcl13, Anxa3, etc. regulated by two or three lncRNAs.

### Competing endogenous RNA (ceRNA) analysis of DElncRNAs, miRNAs and DEmRNAs

**Fig. 7:**
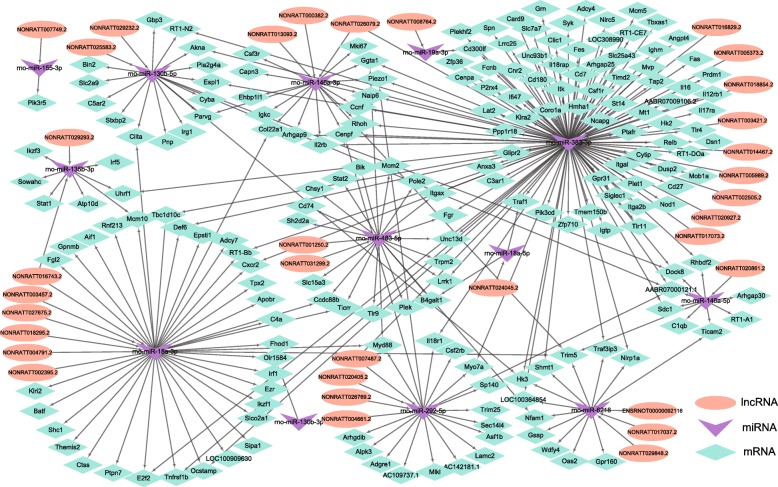
CeRNA network constructed by 34 DElncRNA nodes, 13 miRNA nodes and 192 DEmRNA nodes with 287 edges. The ellipse node indicates lncRNA, while the arrow node indicating miRNA, and the rhombus node indicating mRNA

As lncRNA may affect the mRNA translation by sponging miRNA, the ceRNA network was constructed based on sequence similarity, maximum binding free energy and expression consistency, and 34 DElncRNAs, 13 miRNAs and 192 DEmRNAs with 287 edges were finally involved (Fig. 7). The majority of ceRNA pairs may bind competitively the following miRNA, miR-383-3p, miR-146-3p, miR-146-5p, miR483-5p, miR-292-5p, miR-18a-3p, miR-135b-3p, miR-130b-5p, indicating their core roles in the potential ceRNA regulation mechanisms.

## Discussion

By high-throughput sequencing of the transcriptome, we found 1220 differently expressed mRNAs (DEmRNAs) (1171 up-regulated and 49 down-regulated) and 323 differently expressed lncRNAs (DElncRNAs) (246 up-regulated and 77 down-regulated) in the lumbar spinal cord of BCP rats established by implantation of Walker 256 carcinoma cells into tibia; then through bioinformatic analysis of differently expressed genes, we found that a considerable proportion of them were highly expressed in microglia and extensively involved in immunological and inflammatory processes/pathways and partial DElncRNAs and DEmRNAs may have direct or indirect regulatory relationship.

As previously reported [32–34], the inoculation of Walker 256 breast cancer cells into medullary cavity of tibia could cause significant bone destruction and BCP response in rats, and could induce microglia and astrocyte activation in the spinal cord. As the site of secondary neuron of pain processing, spinal cord dorsal horn integrates and modulates noxious stimuli signal and extensive molecular, cellular and functional changes occur in cancer bone metastasis conditions. LncRNA could regulate coding genes expression and function [35, 36]; however, researches about their roles in BCP are still rare and limited. Given that the biological function of most lncRNA is still unknown, RNA sequencing of lncRNA and mRNA was performed to obtain a global landscape of gene expression alteration in the present study. Furthermore, bioinformatic analyses were conducted to deduce possible functions and regulatory coding genes of DElncRNAs attempting to finding new interventional targets of BCP.

Functional analysis of DEmRNAs and DElncRNAs indicated that they are primarily involved in inflammatory and immunological responses, represented by Class II major histocompatibility complex transactivator (CIITA) and CD74. It has been reported that robust activation of microglia and astrocytes in the spinal cord in BCP model and inhibition of spinal neuroinflammation could significantly attenuate BCP, probably through the means of AMPK activation [37], reactive oxygen species (ROS) scavenging [38], leucine-rich repeat and pyrin domain containing protein 3 (NLRP3) inhibition [39], etc. The central nervous system (CNS) may be not a completely immunological privileged organ especially in certain pathologic conditions; thus CD8^+^ T cell could infiltrate into the spinal cord in BCP [40], maybe by the structural and functional CNS lymphatic vessels [41]. In our differential expression list, CIITA is a key regulator of MHC II expression, which mediates antigen presentation and the reactivation of CNS-infiltrated encephalitogenic T cells in experimental allergic encephalomyelitis [42] and multiple sclerosis [43]. In the study of BCP, Song, Z. et al. has proved CIITA is upregulated by MEK/ERK/STAT1 axis and facilitates MHC II RT1B expression in microglia of spinal cord [44]. Another immune molecule, CD74, as chaperone that regulates antigen presentation for immune response and cell surface receptor for the cytokine macrophage migration inhibitory factor (MIF), is upregulated in our results. While in the study of formalin-induced inflammatory pain, it was reported that CD74 and MIF were both upregulated in the ipsilateral spinal cord dorsal horn [45]. CD74 also participates in sciatic chronic constriction nerve injury-evoked neuropathic pain by activation of ERK1/2-NMDA receptor and/or -PGE2 cascade [46].

We also identified significant upregulation of a series of oxidative stress related genes in BCP, represented by Cytochrome B-245 Beta Chain (CYBB) and Transient Receptor Potential Cation Channel Subfamily M Member 2 (TRPM2). Oxidative stress, which is mainly carried out by reactive oxygen species (ROS) and heightens during inflammation, is involved in the pathogenesis of BCP [38]. As the main sources of ROS in CNS, NADPH oxidase 2 (Nox2) has been proved to play a critical role in nerve injury induced microglial activation in spinal cord and subsequent expression of proinflammatory cytokines in neuropathic pain [47]. As the catalytic subunit of NADPH oxidase complex 2, transcripts of CYBB (gp91phox) are usually used to evaluate the Nox2 level. TRPM2 is a Ca^2+^-permeable nonselective cation channel that is directly activated by free cytosolic ADP-ribose and indirectly activated by ROS such as hydrogen peroxide (H_2_O_2_) [48]. TRPM2 is expressed in peripheral macrophages and spinal microglia and it mediates the pathogenesis of inflammatory and neuropathic pain through the aggravation of pronociceptive inflammatory responses [49]. As a monitor of oxidative stress, TRPM2 is considered as a new target of a spectrum of neuroinflammation associated with CNS disorders, including chronic pain [50].

A few of lncRNAs function have been explored in pain research, such as KCNA2-AS, uc.48+, NONRATT021972, MRAK009713, XIST, CCAT1 [15]; but the function of most lncRNAs remains unknown. We performed GO enrichment and KEGG analysis of potential target genes of DElncRNAs to detect their general biological function, which showed high degrees of consistency with DEmRNAs. Then we constructed co-expression network and ceRNA network to explore the relationship between DElncRNAs and coding genes.

LncRNA could regulate gene expression *in cis*, which influence the expression and/or chromatin state of nearby genes via the sequence-dependent manner (recruiting regulatory factors), transcription and/or splicing of the lncRNA dependent regulatory manner, or functional DNA elements within lncRNA loci [35]. The co-expression network and validated DEmRNAs and DElncRNAs indicated that NONRATT007487.2 may regulate hydroxyl carboxylic acid receptor type 2 (Hcar2) *in cis*, which was recently reported to be involved in neuropathic pain [51]. And CD74 and Trpm2 may be regulated by their adjacent lncRNA shown in the co-expression network. LncRNA could also regulate gene expression *in trans*, which influence gene expression throughout the cell, by regulating chromatin states and distant gene expression (for example, acting as a scaffold), influencing nuclear architecture, directly binding to protein and RNA [35]. The co-expression network and validated DEmRNAs and DElncRNAs suggested NONRATT026544.2 may regulate the expression of Cybb, and NONRATT004661.2 may regulate Ncf1 or ccl9 *in trans*.

LncRNA has been acknowledged to sponge miRNA and therefore protect their target mRNAs from repression, called competing endogenous RNA (ceRNA). Those lncRNAs harboring multiple binding sites of identical miRNA are more likely candidates [52, 53]. CeRNA network hints that the upregulated lncRNA NONRATT007487.2 may interact with rno-miR-292-5p or rno-miR-383-3p, NONRATT004661.2 may interact with rno-miR-292-5p, consequently influencing the levels of the downstream mRNA.

However, we have to recognize that the quantification of RNA-seq could be affected by many factors though we proceeded strict quality control in each major step and the results were confirmed by traditional RT-qPCR. Moreover, gene expression fluctuations measured by RNA-seq could be partially reflected by cell population fluctuations (e.g. increase in microglia population in pathological pain [54, 55]), so it is necessary to perform single cell RNA sequencing (scRNA-Seq) or RNA-seq for a certain cell type (sorted by FACS, for example) to investigate gene expression in individual cell types. In addition, these bioinformatic analyses and predictions are theoretical, limited and maybe even controversial [53], therefore rigorous experimental verification is indispensable to obtain accurate conclusions.

To conclude, the sequencing analysis provides a landscape of dysregulated lncRNA and mRNA in the spinal cord of bone cancer pain; and functional analysis indicates differential expressed genes mainly concentrated in inflammatory and immunologic processes. Our present work may provide some insights and lay a foundation for future research to shed light on the pathogenesis of bone cancer pain.

## Supplementary information

**Additional file 1: Table S1.** Average paw withdrawal mechanical threshold (PWMT) of each animal at each timepoint.

**Additional file 2: Table S2.** Statistical data of high-throughput sequencing for six samples.

**Additional file 3: Figure S1.** Total identified mRNAs and differential expressed mRNAs (DEmRNAs) mapped to the rat genome. Total identified mRNAs were marked by blue lines on the left side of chromosomes and DEmRNAs were marked by blue circles on the right side of chromosomes. The idiogram and GC content background data of rn6 rat genome were from Idiographica (Version 2.4) as illustrated in Materials and Methods.

**Additional file 4: Figure S2.** Total identified lncRNA and differential expressed lncRNA (DElncRNA) mapped to rat genome. Total identified lncRNAs were marked by red lines on the left side of chromosomes and DEmRNAs were marked by red triangles on the right side of chromosomes. The idiogram and GC content background data of the rn6 rat genome were from Idiographica (Version 2.4) as illustrated in Materials and Methods.

**Additional file 5: Table S3.** The raw data of RT-qPCR validation assay.

**Additional file 6: Figure S3.** The expression level of differential expressed mRNAs (DEmRNAs) in seven types of cells in central nervous system. Expression heatmap was constructed by our DEmRNA list and relative expression data from research of Ye Zhang et al. as illustrated in Materials and Methods. FPKM, Fragments Per Kilobase per Million.

**Additional file 7: Table S4.** Top 20 clusters with their representative enriched terms (one per cluster).

## Data Availability

Please contact the author for data requests.

## Abbreviations

BCP: Bone cancer pain
ceRNA: Competing endogenous RNA
DElncRNAs: Differently expressed lncRNAs
DEmRNAs: Differently expressed mRNAs
FDR: False discovery rate
GO: Gene Ontology
KEGG: Kyoto Encyclopedia of Genes and Genomes
lncRNAs: Long non-coding RNAs
PCC: Pearson correlation coefficient
PWMT: Paw withdrawal mechanical threshold
ROS: Reactive oxygen species
RT-qPCR: Real-time quantitative PCR
