# Supplementary figures and images for: Transcriptomic analysis of long noncoding RNAs and mRNAs expression profiles in the spinal cord of bone cancer pain rats
Source: Mol Brain. 2020 Mar 24;13:47. doi: 10.1186/s13041-020-00589-2 (PMC7092675; doi:10.1186/s13041-020-00589-2)

mRNA  
DEmRNA

GC content  
High

Low

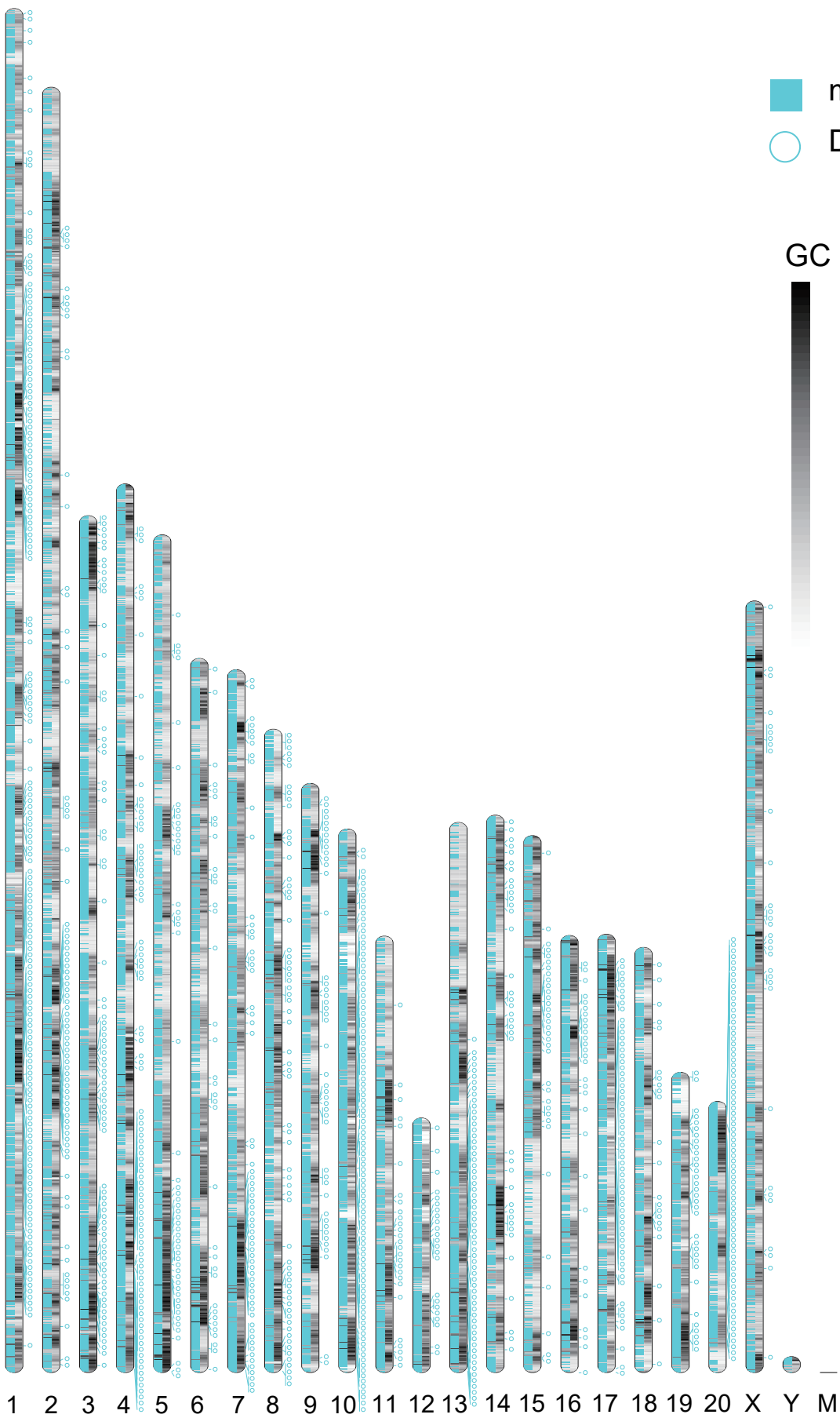

Supplement: Supplementary file 3 — Additional file 3: Figure S1. Total identified mRNAs and differential expressed mRNAs (DEmRNAs) mapped to the rat genome. Total identified mRNAs were marked by blue lines on the left side of chromosomes and DEmRNAs were marked by blue circles on the right side of chromosomes. The idiogram and GC content background data of rn6 rat genome were from Idiographica (Version 2.4) as illustrated in Materials and Methods. [file 13041_2020_589_MOESM3_ESM.pdf]

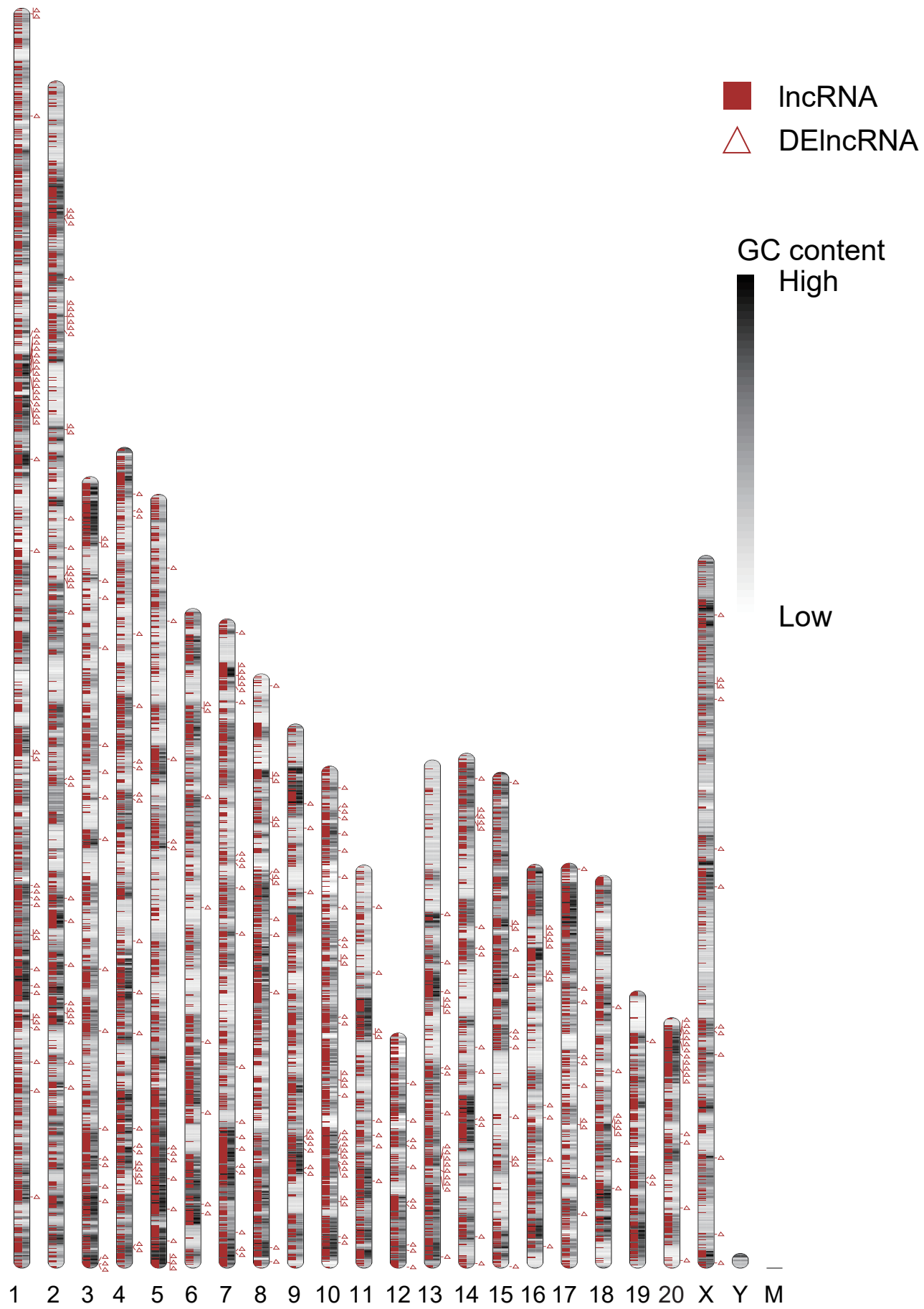

Supplement: Supplementary file 4 — Additional file 4: Figure S2. Total identified lncRNA and differential expressed lncRNA (DElncRNA) mapped to rat genome. Total identified lncRNAs were marked by red lines on the left side of chromosomes and DEmRNAs were marked by red triangles on the right side of chromosomes. The idiogram and GC content background data of the rn6 rat genome were from Idiographica (Version 2.4) as illustrated in Materials and Methods. [file 13041_2020_589_MOESM4_ESM.pdf]

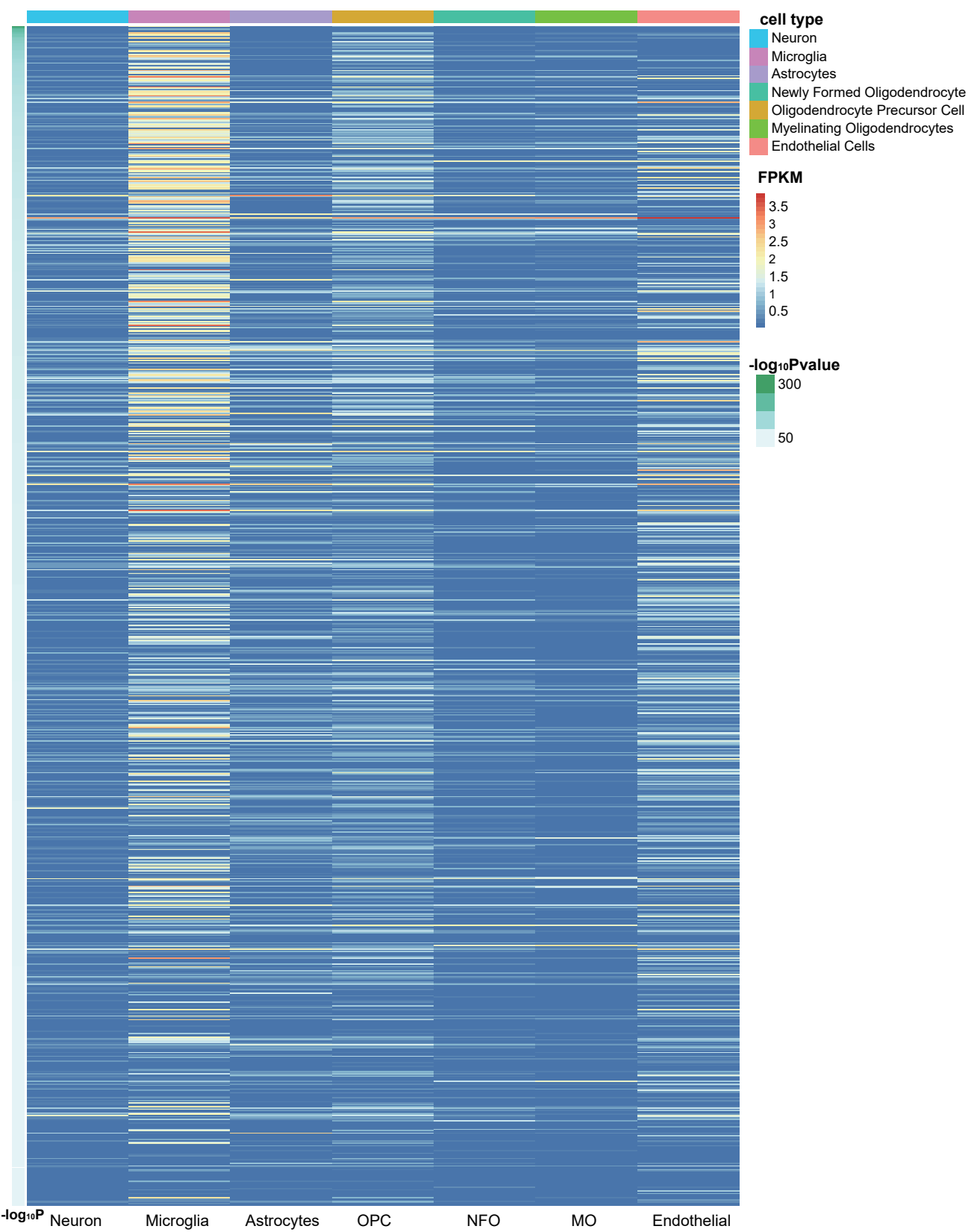

Supplement: Supplementary file 6 — Additional file 6: Figure S3. The expression level of differential expressed mRNAs (DEmRNAs) in seven types of cells in central nervous system. Expression heatmap was constructed by our DEmRNA list and relative expression data from research of Ye Zhang et al. as illustrated in Materials and Methods. FPKM, Fragments Per Kilobase per Million. [file 13041_2020_589_MOESM6_ESM.pdf]
